# The impact of pre‐existing influenza antibodies and inflammatory status on the influenza vaccine responses in older adults

**DOI:** 10.1111/irv.13172

**Published:** 2023-07-12

**Authors:** Min Kang, Fangmei Lin, Zhanpeng Jiang, Xiaohua Tan, Xia Lin, Zaolan Liang, Cheng Xiao, Yonghe Xia, Wenda Guan, Zifeng Yang, Guangchuang Yu, Mark Zanin, Shixing Tang, Sook‐San Wong

**Affiliations:** ^1^ School of Public Health Southern Medical University Guangzhou P. R. China; ^2^ Guangdong Center for Disease Control and Prevention Guangzhou P. R. China; ^3^ Guangzhou Medical University, Xinzao Guangzhou P. R. China; ^4^ State Key Laboratory for Respiratory Diseases and National Clinical Research Centre for Respiratory Disease Guangzhou P.R. China; ^5^ HKU‐Pasteur Research Pole, School of Public Health, LKS Faculty of Medicine The University of Hong Kong Hong Kong China; ^6^ Zhongshan Yiyan Bio‐Pharmaceutical Co., Ltd Zhongshan P. R. China; ^7^ Guangzhou Institute of Respiratory Health First Affiliated Hospital of Guangzhou Medical University, National Center for Respiratory Medicine Guangzhou P.R. China; ^8^ Department of Bioinformatics, School of Basic Medical Sciences Southern Medical University Guangzhou P. R. China; ^9^ School of Public Health, LKS Faculty of Medicine The University of Hong Kong Hong Kong China; ^10^ Centre for Immunology & Infection Shatin Hong Kong

**Keywords:** antibodies, cytokines, influenza, older adults, vaccines

## Abstract

Age‐associated immune changes and pre‐existing influenza immunity are hypothesized to reduce influenza vaccine effectiveness in older adults, although the contribution of each factor is unknown. Here, we constructed influenza‐specific IgG landscapes and determined baseline concentrations of cytokines typically associated with chronic inflammation in older adults (TNF‐α, IL‐10, IL‐6, and IFN‐γ) in 30 high and 29 low influenza vaccine responders (HR and LR, respectively). In a background of high H3 antibody titers, vaccine‐specific H3, but not H1, antibody titers were boosted in LRs to titers comparable to HRs. Pre‐vaccination concentrations of IL‐10 were higher in LRs compared with HRs and inversely correlated with titers of pre‐existing influenza antibodies. Baseline TNF‐α concentrations were positively correlated with fold‐increases in antibody titers in HRs. Our findings indicate that baseline inflammatory status is an important determinant for generating post‐vaccination hemagglutinin‐inhibition antibodies in older adults, and IgG responses can be boosted in the context of high pre‐existing immunity.

## 1 BACKGROUND

Vaccination is recommended as the most effective way to prevent influenza disease and its associated complications, particularly for vulnerable populations such as older adults. However, influenza vaccination has been found to be less efficacious in this population.^1^ Immunosenescence, the age‐associated decline in immunity, and frailty, a geriatric syndrome characterized by increased vulnerability to adverse health outcomes and multi‐system dysregulation,^2^, ^3^, ^4^ have both been linked to suboptimal vaccine responses.^5^, ^6^ A physiological feature underlying frailty is “inflammaging,” the state of chronic, low‐grade inflammation associated with aging that can be characterized by elevated concentrations of cytokines such as C‐reactive protein (CRP), interleukin (IL)‐6, tumor necrosis factor (TNF)‐ α, and IL‐10.^7^, ^8^

Pre‐existing influenza immunity can also influence antibody responses after influenza vaccination, and, with age, increasing numbers of influenza virus exposures can result in complex immunological profiles that may be detrimental to generating immunity against new viruses.^9^, ^10^ For example, older adults, generally defined as those older than 65 years of age, have proportionately more antibodies that target influenza virus conserved epitopes than any other age group.^11^, ^12^ However, these antibodies have poorer virus neutralization capacity, which may contribute to reduced vaccine effectiveness.^10^ Indeed, in some studies, pre‐existing immunity appears to be a more important determinant of vaccine responses than immunosenescence in older adults.^13^, ^14^, ^15^

While influenza vaccine‐induced antibody responses are conventionally measured using the hemagglutination‐inhibition (HI) assay, we studied pre‐existing immunity by quantifying antigen‐specific IgG titers. The HI assay detects antibodies targeting the globular head of the influenza virus hemagglutinin (HA) protein, with titers of ≥1:40 being the currently accepted standard for seroprotection as it is associated with a 50% reduction in infection risk in a healthy adult population.^16^, ^17^ HI‐antibodies are typically high‐affinity and antigen‐specific; however, they are not elicited in high titers, and their detection provides only limited sensitivity in quantifying antibody responses. Antigen‐specific IgG antibodies, in contrast, encompass a broader class of binding antibodies that includes HI‐antibodies, are less strain‐specific and are present at high titers. Thus, quantification of antigen‐specific IgG antibodies offers greater sensitivity in detecting changes in the post‐vaccination immune response compared to HI antibodies^18^, ^19^ and may also reflect the underlying mechanism that is perturbed.

Here, using samples collected from an older adult vaccination campaign conducted in Guangdong province in southern China in late 2018, we evaluated the influence of pre‐existing influenza A virus (IAV) antibodies and immune status on vaccine responses in older adults. The HA protein of IAV subtypes can be grouped into two major phylogenetic groups: Group 1, comprising subtypes H1, H2, H5, H6, H8, H9, H11, H12, H13, H16, H17, and H18; and Group 2, comprising subtypes H3, H4, H7, H10, H14, and H15. To capture the overall HA IgG antibody response, including cross‐reactive antibodies, we measured the IgG response against a panel of HA proteins from both groups. To capture the overall HA IgG antibody response, including cross‐reactive antibodies, we measured the IgG response against a panel of HA proteins from both groups. We also measured serum cytokine concentrations and performed correlative analyses to identify the relationship between these two factors and study the roles of immune status and pre‐existing immunity in influenza vaccine immunogenicity in older adults.

## 2 METHODS

### 2.1 Ethics approval

The study was approved by the Ethics Review Committee of the Guangdong Center for Disease Control and Prevention, China (GDCDC) (No. 2018023). Participants provided written consent, and the data were anonymized for analyses.

### 2.2 Study design

Serum samples were collected from older adults who were part of a vaccination campaign conducted by the GDCDC in December 2018. Five hundred three participants received a standard dose of the 2018–2019 quadrivalent inactivated vaccine (Hualan Biologics), comprising 15 μg each of the Northern Hemisphere vaccine strains: A/Michigan/45/2015 (H1N1), A/Singapore/INFIMH‐16‐0019/2016 (H3N2), B/Colorado/06/2017 (Victoria lineage), and B/Phuket/3073/2013 (Yamagata lineage). Serum samples were collected at baseline (day zero [D0]) and approximately 30 days (D30) after vaccination. Because of limited antigen availability for influenza B for the generation of antibody landscapes, we focused our study on IAV responses.

We initially selected 30 high‐responders (HR) and 30 low‐responders (LR) based on their post‐vaccination HI‐antibody response to the IAV vaccine strains; however, one LR sample was eventually excluded due to insufficient sera. This sample size was based on our previous study evaluating differences in the immune profile associated with seroconversions.^20^ HRs showed at least a four‐fold increase in HI for both IAV vaccine strains post‐vaccination, while LRs did not. To minimize the influence of pre‐existing homologous antibodies, we selected participants with baseline HI‐titers ≤1:40, but this was only possible for antibodies against subtype H1N1 IAVs due to high pre‐existing HI‐titers for subtype H3N2 IAVs.

### 2.3 HI assay

Sera pre‐treated with receptor‐destroying enzyme (RDE) and titrated in serial two‐fold dilutions starting at 1:10 were tested against the four vaccine strains. Reference antigens were propagated in eggs and prepared according to standard protocol.^21^ HI‐antibody titers were determined as the reciprocal of the highest dilution causing complete HI of 0.5% chicken erythrocytes.

### 2.4 Enzyme‐linked lectin assay (ELLA)

The ELLA was used to measure neuraminidase (NA)‐specific antibodies using recombinant reverse‐genetic viruses expressing the NA of the target strains and a mismatched HA, as previously described.^22^ Paired sera were tested together in the same run. NI‐titers were measured as the reciprocal of the highest dilution that resulted in more than 50% signal inhibition compared to virus‐only wells. A sera with previously pre‐defined titers against the reference antigens was used as an internal control.

### 2.5 Enzyme‐linked immunosorbent assay (ELISA)

Recombinant HA proteins representing a broad array of Groups 1 and 2 avian and human influenza virus strains (Sinobiologicals, Table S3) were coated at 0.5 μg/mL onto 96‐well high‐binding immunoassay plates in coating buffer overnight at 4°C. Negative wells were coated with buffer only. ELISA was performed as previously described.^20^ To control for inter‐assay variability, sera from a healthy adult with pre‐defined titers against five reference antigens in this panel derived from three independent assays was used as an internal standard. Paired sera were run in the same run, and each run consisted of similar numbers of HR and LR samples. IgG titers for the influenza B strains were not measured due to the limited availability of influenza B HA proteins.

### 2.6 Cytokine quantification

Cytokine concentrations in serum samples for TNF‐α, IFN‐γ, IL‐6, and IL‐10 were measured using the human high‐sensitivity cytokine premixed Luminex assay (R&D Biosystems) according to the manufacturer's instructions and analyzed using the Bioplex Manager software (refer to Table S4 for standard ranges and limits of detection [LoD]). As concentrations lower than the minimum standard used (i.e., lower limit of quantitation [LLoQ]) were less accurate, we normalized these data to avoid artificially inflating the effect size. All samples of LLoQ were assigned the median value of all LLoQ data.

### 2.7 Statistical analysis

Categorical data were analyzed by Fisher's exact test. For antibody data, samples that did not meet the assay detection threshold were assigned a value equal to half of the starting dilutions. Antibody data were log‐transformed to calculate geometric mean titers (GMTs). The titer differences between paired samples and groups were analyzed by paired and unpaired t‐tests, respectively, using two‐tailed tests of significance and Bonferroni's post‐test. Log‐transformed area‐under‐the‐curve (AUC) data were used for comparisons among groups using t‐tests with Bonferroni's corrections where applicable (GraphPad Prism version 8).

Correlation analyses were performed using Pearson's method (R version 4.0.5), with p‐values adjusted by controlling for the false discovery rate using the Benjamini‐Hochberg method. K‐means cluster analysis was applied to identify groups of individuals with similar HA‐IgG titers at D0. The optimal number of clusters was determined using NbClust.^23^ To generate HA‐IgG landscapes, amino acid sequence distance among HAs was calculated using the substitution matrix of BLOSUM62. Antibody landscapes were generated using geometric means of HA‐IgG titers using multilevel B‐splines. The 3D plots were generated by Plotly in R.

## 3 RESULTS

### 3.1 Serologic profiles of vaccine HR and LR

We used pre‐ and post‐vaccination sera samples from 30 HR and 29 LR that received the 2018–2019 quadrivalent inactivated influenza vaccine. Although age and sex‐matched groups were initially planned, females were slightly over‐represented in the LR in the final selected cohort (Table 1). There were no other significant differences in age distribution, smoking status, or co‐morbidities between LR and HR.

**TABLE 1:** Demographic and characteristics of the participants in the study.

Characteristics	High‐responders (*n* = 30)	Low‐responders (*n* = 29)	*p*‐value^a^
Gender, *n* (%)			
male	29 (96.7)	22 (75.9)	*0.03*
female	1 (3.3)	7 (24.1)	
Age, median (IQR), years old	69 (67,73)	70 (68,73)	0.42
60–69 years old, *n* (%)	15 (50.0)	15 (51.7)	0.90
≥70 years old, *n* (%)	15 (50.0)	14 (48.3)	
Current smoker, *n* (%)	15 (50.0)	9 (31.0)	0.14
Comorbidities, *n* (%)			
Chronic respiratory disease	10 (33.3)	7 (24.1)	0.44
Hypertension	4 (13.3)	7 (24.1)	0.29
Diabetes	2 (6.7)	0 (0)	0.49
Cardiovascular disease	2 (6.7)	2 (6.9)	1.00

^a^ *p*‐values were derived from Fisher's exact tests, with *p* < 0.05 being italicized.

**FIGURE 1:**
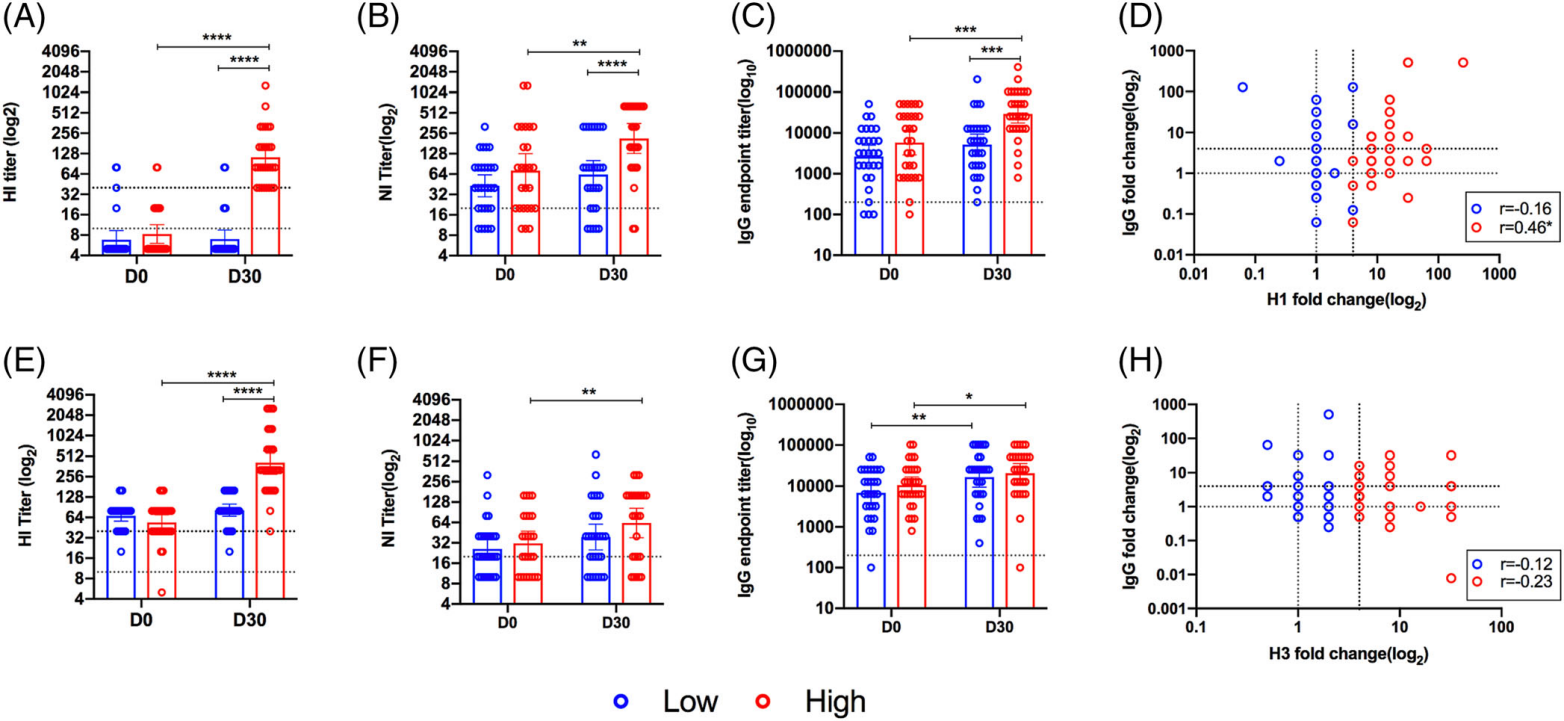
Serologic profile against the vaccine components in the high (*n* = 30, red) and low (*n* = 29, blue) vaccine responders at baseline (day zero [D0]) and at day 30 (D30) post‐vaccination. Antibody titers against the influenza A subtype (A–D) A/H1N1 and (E–H) A/H3N2 vaccine strains as measured by (A, E) hemagglutination inhibition (HI) assay, (B, F) neuraminidase inhibition (NI) assay, and (C, G) hemagglutinin (HA)‐specific IgG enzyme‐linked immunosorbent assay (ELISA) using recombinant HA protein. (D, H) Correlation between HA‐IgG titer fold change with HI‐titer fold change and r indicates the Pearson’s correlation coefficient. * indicates *p* < 0.05, ** indicates *p* < 0.01, and *** indicates *p* < 0.001 by paired t‐test for D0 vs. D30 comparisons and by one‐way ANOVA with Bonferroni's corrections for cross‐group comparisons.

With the exception of H3N2, pre‐vaccination HI titers were ≤1:40 to all other vaccine‐strains. There were no significant differences in the HI GMTs between HRs and LRs to any vaccine components at baseline (Figure 1A,E, Table 2). Post‐vaccination HI‐antibody titers in the HRs increased by 7.7 to 13.6‐fold compared with 1.0 to 1.7‐fold in the LRs (Table 2). Notably, despite the absence of a four‐fold increase, LRs still showed a marginally significant HI‐GMT increase in subtype H3N2 IAVs and significant GMT increases in the influenza B virus vaccine components. Ten percent and 14% of LRs also seroconverted to the influenza B/Victoria and B/Yamagata vaccine strains, respectively. However, the average geometric mean fold change and seroconversion rate, defined as a four‐fold increase in antibody titers, in the LRs were lower than the HRs by about five to six folds.

**TABLE 2:** Antibody responses to vaccine components in high‐ and low‐responders as measured by the hemagglutination‐inhibition (HI) assay. Antibody titers were expressed as geometric mean titers (GMTs), and fold changes between day 0 (GMT 1) and day 30 (GMT 2) were expressed as geometric mean fold change (GMFC). Seroconversions (SC) refer to the percentage of vaccinees that achieved at least a four‐fold increase in antibody titers after vaccination at day 30. Statistically significant *p*‐values (<0.05), adjusted for multiple comparisons, are italicized, while ns denotes no significance.

Groups	Antibody titer	N	H1			H3			BV			BY		
			HI	95% CI	*p*‐value	HI	95% CI	*p*‐value	HI	95% CI	*p*‐value	HI	95% CI	*p*‐value
Low	GMT 1	29	7	5.0–9.3		68	56.4–81.2		5.635	4.7–6.7		7	5.2–8.9	
	GMT 2		7	5.2–9.5	0.865^a^	82	66.8–100.6	0.058^a^	10	7.0‐13.5	*0.009* ^a^	12	7.5‐17.8	*0.003* ^a^
	GMFC		1.0	0.8–1.4		1.2	1.0–1.5		1.7	1.2–2.6		1.7	1.2–2.3	
	SC percentage		0			0			10			14		
High	GMT 1	30	8	6.0–11.4	ns^c^	54	41.7–70.1	ns^c^	5	5.0–5.0	ns^c^	6	5.0–8.4	ns^c^
	GMT 2		113	80.1–160.0	*<0.0001* ^b^ ^,^ ^d^	413	278.4–611.4	*<0.0001* ^b^ ^,^ ^d^	44	26.2–73.5	*<0.0001* ^b^ ^,^ ^d^	19	34.4–101.9	*<0.0001* ^b^ ^,^ ^d^
	GMFC		13.6	9.5–19.6	*<0.0001* ^e^	7.7	5.6–10.4	*<0.0001* ^e^	8.8	5.2–14.7	*<0.0001* ^e^	9.2	5.0–16.8	*<0.0001* ^e^
	SC percentage		100			100			60			93		

^a^ GMT 2 versus GMT 1 by t‐test in low‐responders.

^b^ GMT 2 versus GMT 1 by t‐test in high‐responders.

^c^ GMT 1, high versus low by t‐test using log‐transformed data.

^d^ GMT 2, high versus low by t‐test using log‐transformed data.

^e^ GMFC, high versus low by t‐test using log‐transformed data.

As inactivated influenza vaccines can contain residual NA proteins that can elicit an antibody response (21), we also measured the NA‐specific antibodies using ELLA. No differences were detected at baseline between the HRs and LRs (Figure 1B,F, Table S1). After vaccination, NI‐antibody GMT increased three‐ and two‐fold against NA subtypes N1 and N2, respectively, in HRs, compared with 1.5‐fold against both subtypes in LRs. Ten (34%) and seven (24%) LRs seroconverted to N1 and N2, respectively. In contrast, 50% and 35% of HRs seroconverted to N1 and N2, respectively.

HRs and LRs showed significant post‐vaccination increases of similar magnitudes in IgG titers to the H3N2 vaccine strain (Figure 1C,G, Table S2). HRs showed an average five‐fold increase in IgG titers against the H1N1 vaccine strain, compared with only 1.9‐fold in LRs. A positive correlation between HA‐IgG and HI‐antibody fold changes was observed in HRs for H1N1, but not for H3N2 vaccine strains (Figure 1D,H).

Taken together, these data indicate that LRs were able to elicit some vaccine‐associated antibody responses, particularly to H3N2 and influenza B viruses, but at lower magnitudes compared to HRs. Despite the absence of significant increases in HI‐antibody titers in H3N2 strains, LRs were still able to produce H3‐specific IgG antibodies.

### 3.2 LR had less Group 1 HA‐IgG antibodies at baseline and showed minimal induction of HA‐IgG antibodies post‐vaccination

**FIGURE 2:**
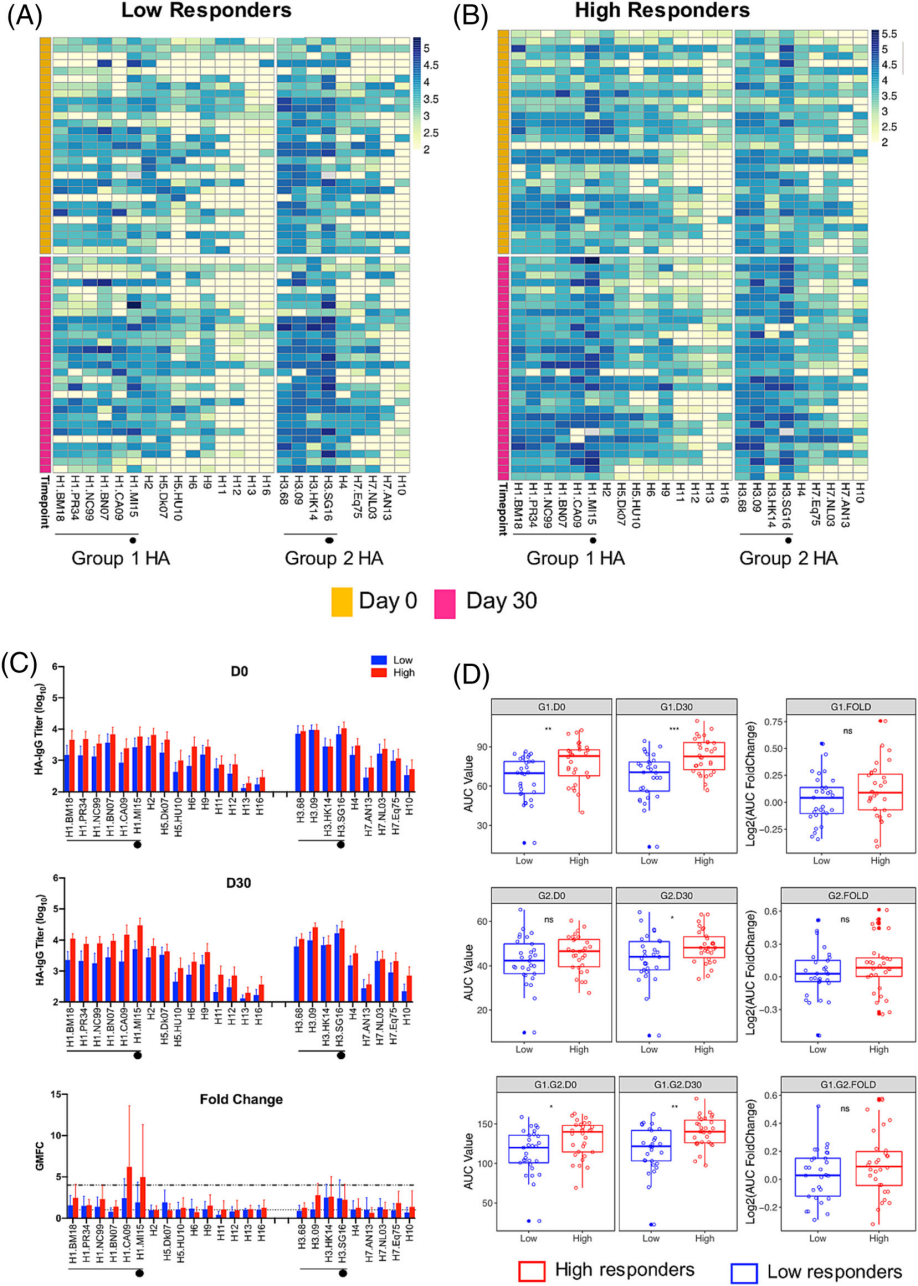
Breadth of influenza hemagglutinin (HA)‐specific IgG reactivity of the high (HR, *n* = 30) and low (LR, *n* = 29) vaccine responders at baseline and after vaccination. Heatmap of log‐transformed IgG titers against 14 recombinant HA proteins (recHA) from Groups 1 and 8 recHA from Group 2 influenza A viruses for the (A) LR and (B) HR. (C) Geometric mean IgG titers at baseline (day zero [D0]) and day 30 (D30) and the geometric mean antibody fold change (GMFC) between D0 and D30 against all tested recHA for HR (red) and LR (blue). Error bars indicate the 95% confidence intervals. (D) Aggregated area‐under‐the‐curve (AUC) index for HR and LR against Group 1 (G1), Group 2 (G2), and combined (Groups 1 and 2, G1.G2) HA IgG reactivity at D0 and D30. Boxplots indicate the median and interquartile range (IQR) of log‐transformed data. HA from human strains was indicated with black lines, while HA from the vaccine strain was indicated with black circles. * indicates *p* < 0.05, ** indicates *p* < 0.01, and ns indicates no significance, by t‐test using log‐transformed data. The full names of virus strains are provided in Table S3.

To examine if pre‐existing IAV HA antibodies contributed to the observed vaccination responses, we measured HA‐reactive IgG antibodies against a panel of recombinant HAs broadly representative of Group 1 and Group 2 IAVs. Reactivity profiles were similar between LRs and HRs, with the majority of the IgG responses directed against human IAV HAs. Some responses were also observed in approximately half of the participants against HAs of the avian subtypes H5, H9, and H7 (Figure 2A,B). At baseline, LRs tended to have lower IgG‐GMTs against all the HAs, except for H3, compared with HRs (Figure 2C). Cumulative AUC data, which is a metric used to sum up HA‐IgG reactivities, showed that, on average, LRs had less total HA‐IgG compared with HRs at baseline, which was primarily driven by lower antibody titers against Group 1 IAV HAs (Figure 2D).

**FIGURE 3:**
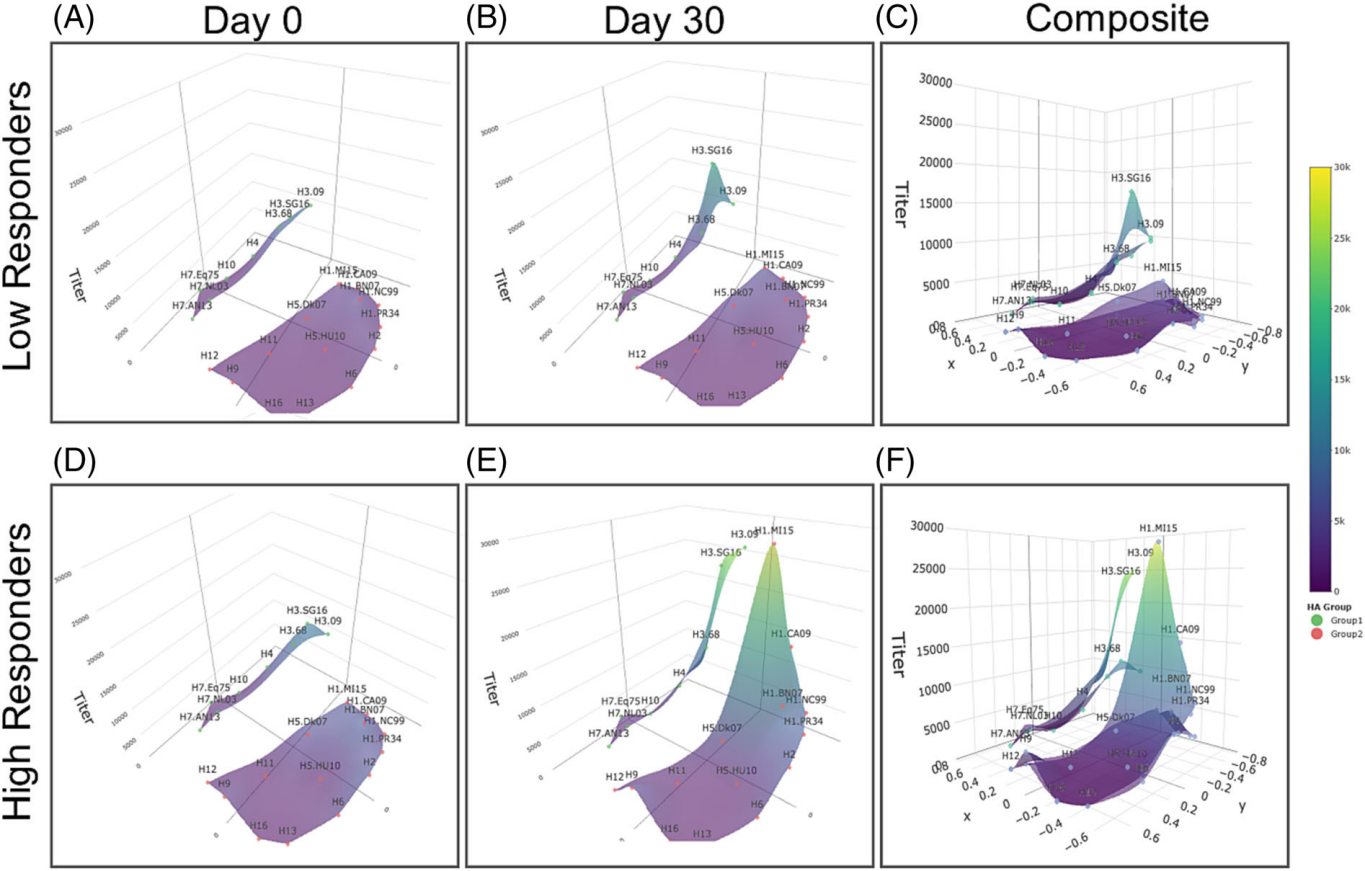
Influenza hemagglutinin (HA) IgG landscapes of the high (HR, *n* = 30) and low (LR, *n* = 29) vaccine responders. IgG titers were measured against 14 recombinant HA proteins (recHA) from Groups 1 and 8‐recHA from Group 2 and visualized as a landscape. HA‐IgG landscape for (A, B) low and (D, E) high responders at day 0 (D0) and day 30 (D30). A composite of D0 and D30 responses is as shown in (C) and (F). The full names of virus strains are provided in Table S3.

Post‐vaccination, the largest IgG increase was detected against the H1 vaccine strain and its antigenic predecessor. AUC was significantly higher in HRs compared with LRs for both Group 1 and 2 IAV antigens, although, notably, no significant differences in AUC fold change were found. Antibody landscapes of HRs showed significant peaks post‐vaccination corresponding to the H1 and H3 IAV vaccine strains and closely related antigens, while the IgG landscape of LRs only showed a modest peak corresponding to the H3 IAV antigens (Figure 3). In summary, these data indicate that (i) post‐vaccination responses were mainly directed against vaccine antigens and closely related strains, and (ii) despite no significant differences detected in the vaccine‐specific IgG titers between LRs and HRs at baseline, LRs had less HA‐IgG antibodies to other H1 strains in the landscape, amounting to less Group 1‐reactive HA antibodies.

### 3.3 Impact of pre‐existing influenza antibodies on subsequent antibody responses

**FIGURE 4:**
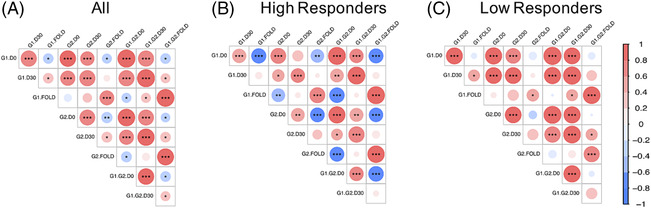
Relationship between pre‐existing antibodies and post‐vaccination responses. Correlation between the baseline (day zero, [D0]) and day 30 (D30) total HA‐IgG area‐under‐the‐curve (AUC) and antibody fold change (FOLD) for Group 1 (G1), Group 2 (G2), and combined (G1.G2) response shown for (A) all groups, (B) high responders, and (C) low responders. The color scale and size of the circle are proportional to the Pearson's correlation; the r‐value and asterisks indicate statistical significance after adjustment for the false discovery rate using the Benjamini–Hochberg method. * indicates *p* < 0.05, ** indicates *p* < 0.01, and *** indicates *p* < 0.001.

We next studied the relationship between pre‐existing influenza antibodies, represented by the AUC metric of the total HA‐IgG, and subsequent vaccine responses. In the pooled analyses, the D0 AUC was positively correlated with the D30 AUC for Groups 1 and 2 HAs but was inversely correlated with antibody fold change (Figure 4A). This relationship was largely driven by responses in HRs, as they showed the same trend but with stronger statistical support in the subgroup analysis (Figure 4B). In contrast, a strong positive correlation between the D0 and D30 AUCs for Groups 1 and 2 HAs was present in LRs (Figure 4C), but no associations with antibody fold change were found, likely due to the small magnitude of the antibody fold changes observed. There was also a positive correlation in the antibody fold–changes between Groups 1 and 2 in both HRs and LRs, suggesting that antibodies were elicited in both HA groups concurrently. Taken together, our data indicates high titers of pre‐existing antibodies were associated with minimal boosting, with the largest antibody increases associated with the lowest pre‐existing titers.

**FIGURE 5:**
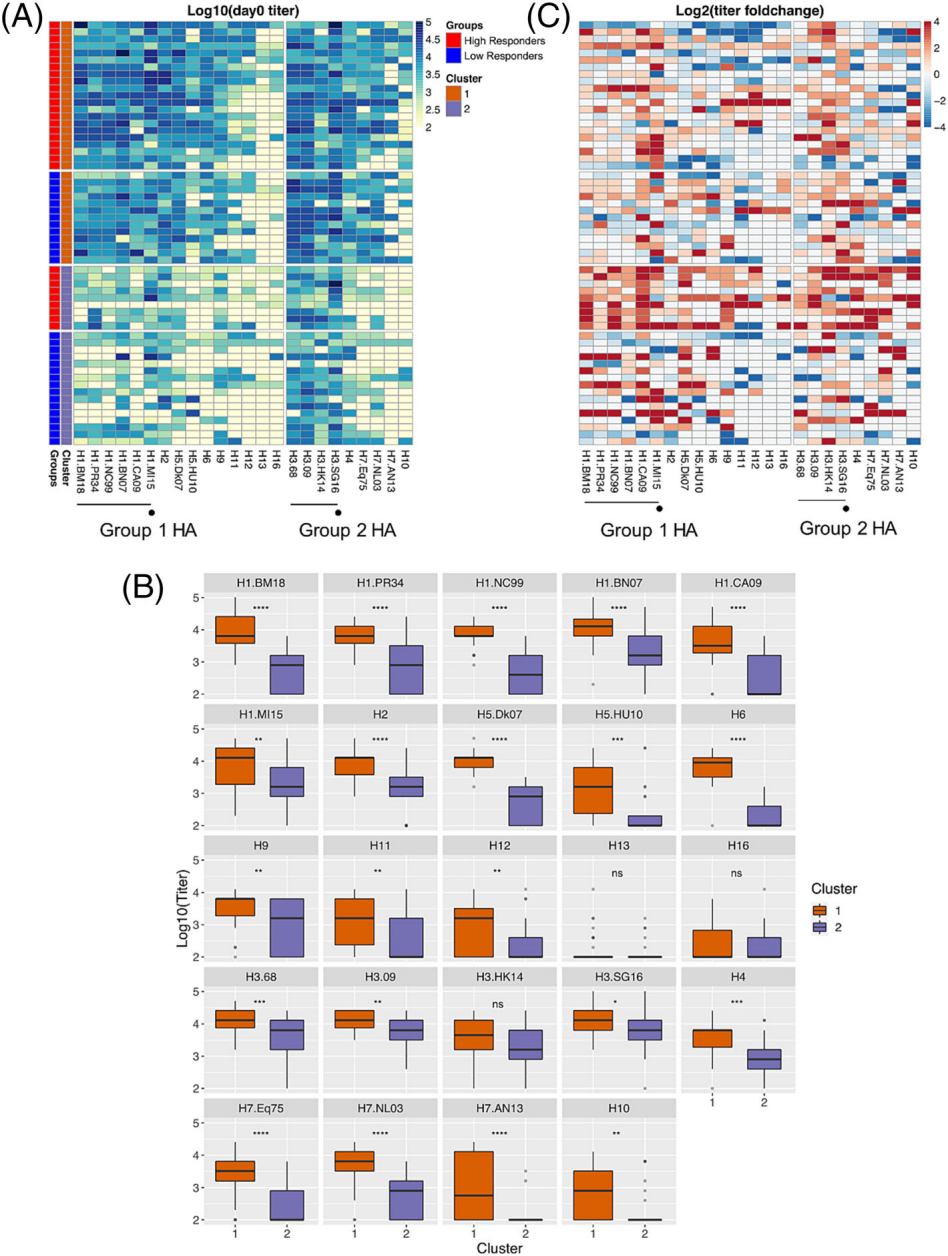
Baseline reactivity profile for the high (HR, *n* = 30) and low (LR, *n* = 29) vaccine responders. (A) Unsupervised clustering of the HR and LR baseline reactivity profiles using k = 2. (B) Boxplots indicate the median and interquartile range (IQR) of log‐transformed data for each strain for Clusters 1 and 2. (C) Antibody titer fold change according to clusters and response groups as shown in (A). Black lines indicate the median and interquartile range (IQR) of log‐transformed data. HA from human strains were indicated with black lines, while HA from the vaccine strain was indicated with black circles. * indicates *p* < 0.05, ** indicates *p* < 0.01, and ns indicates no significance, by t‐test using log‐transformed data.

To determine if the baseline reactivity of pre‐existing antibodies was different between HR and LR, we performed unsupervised k‐mean clustering of the HA‐IgG profiles at D0 into two optimized clusters (Figure S1). Cluster 1 was comprised of individuals who had high IgG titers to most HA proteins, while Cluster 2 was comprised of individuals who had undetectable to moderate IgG titers to all the HA proteins (Figure 5A). The biggest differences were observed in the aggregate titers against HA proteins from historical human subtype H1 IAVs and avian HAs (Figure 5B). Clusters 1 and 2 consisted of 21 and nine HR and 13 and 16 LR, respectively (Figure 5). This non‐random distribution (p = 0.045) supported the observation that individuals with more pre‐existing antibodies, in terms of magnitude and breadth of reactivity, were likely to be HR. Post‐vaccination, HR in Cluster 2 showed the strongest and broadest increases in antibody titers (Figure 5C), in agreement with our previous observation that pre‐existing antibodies at lower titers were boosted more significantly than those at higher titers after vaccination.

### 3.4 LR had higher levels of IL‐10 compared with HR

Given the lower baseline titers of IAV‐specific antibodies in LRs compared with HRs, we sought to determine if LRs were in a state of relative immune suppression. We measured the baseline serum concentrations of four major cytokines associated with age‐associated inflammation: TNF‐α, interferon (IFN)‐γ, and IL‐6, which are pro‐inflammatory, and IL‐10, which has immune‐suppressive functions. Of 58 serum samples, 56 yielded data in the bead‐based assay (Table S4). Fifty‐five (98%) had IFN‐γ concentrations LLoQ. Eleven (20%), 23 (41%), and 21 (38%), respectively, of the samples had TNF‐α, IL‐6, and IL‐10 concentrations LLoQ, respectively. As LLoQ data is less reliable, we excluded IFN‐γ from further analyses and normalized the LLoQ data in the remaining dataset to minimize inflating their effect during subsequent analyses.

**FIGURE 6:**
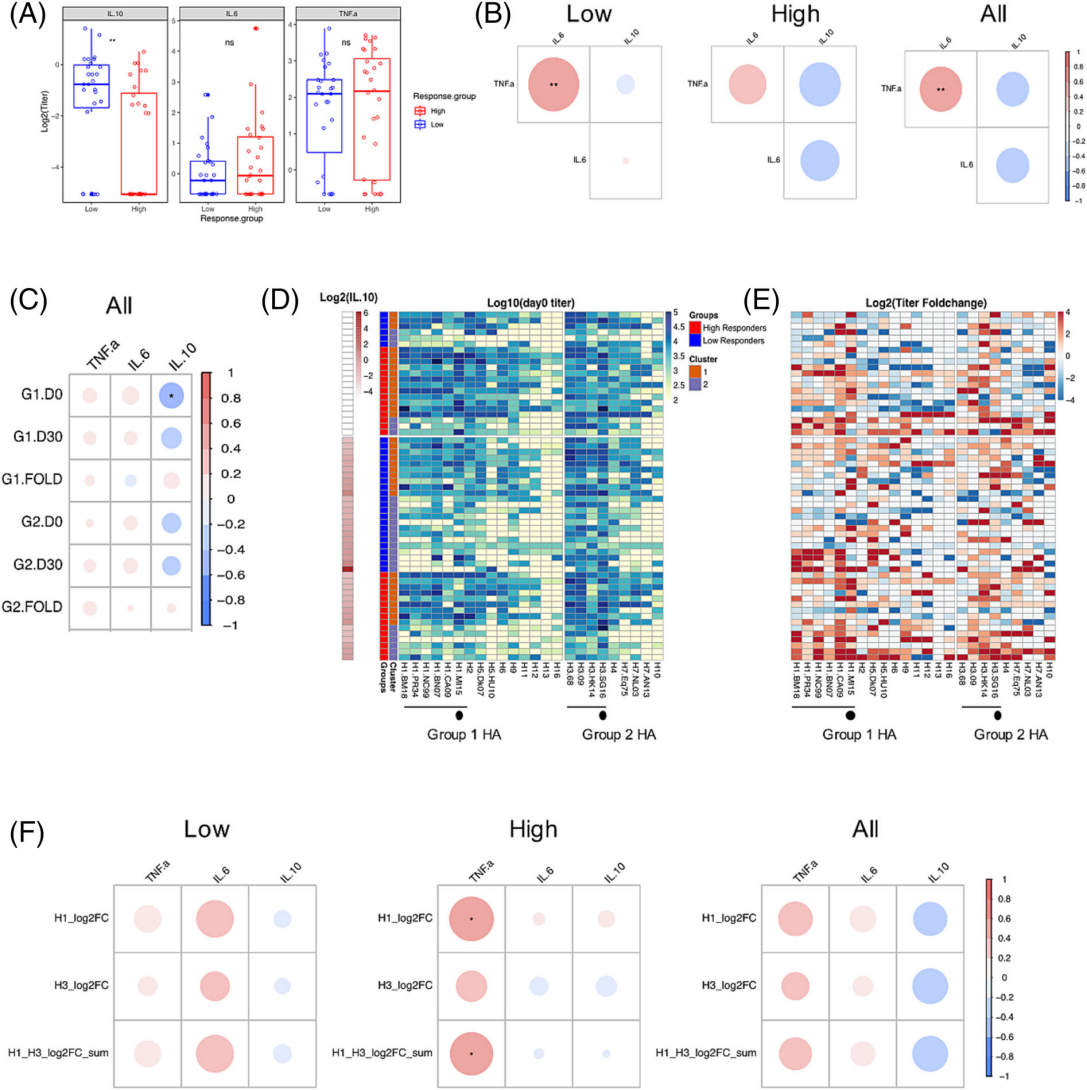
Relationship between the cytokine profile and influenza vaccine responses. (A) Cytokine concentrations of IL‐10, IL‐6, and TNF‐α of the high (*n* = 28) and low (*n* = 27) vaccine responders at baseline. Boxplots indicate the median and interquartile range (IQR) of log‐transformed data. (B) Correlation among cytokine concentrations in high, low, and all vaccine responders. (C) Correlation for the cytokine concentrations with the hemagglutinin (HA)‐IgG profile based on the Group 1 (G1) and Group 2 (G2) AUC at day 0 (D0) and day 30 (D30) and their respective fold changes (FOLD) for low and high responders. (D) HA‐IgG profile as grouped by IL‐10 concentrations, reactivity clusters, and response groups; and (E) its associated fold change, grouped as in (D). (F) Correlation between the cytokines' concentrations and the log‐fold change (logFC) of the hemagglutination‐inhibition (HI) titers against A/H1N1, A/H3N2, and to A/H1N1 and A/H3N2 (H1 + H3). One low responder with an outlying IL‐10 signal was excluded from analysis, except in (D) and (E). The color scale and the size of the circle in (B, C–F) are proportional to the Pearson’s correlation; the r‐value and asterisks indicate statistical significance after adjustment for the false discovery rate using the Benjamini–Hochberg method. * indicates *p* < 0.05, ** indicates *p* < 0.01, and *** indicates *p* < 0.001.

Average concentrations of IL‐10 at baseline were higher in LRs compared with HRs (p = 0.0068, Figure 6A), while no statistically significant differences in TNF‐α and IL‐6 concentrations were detected between LR and HR. Concentrations of IL‐6 were positively correlated with TNF‐α, particularly in LRs (p = 0.0045, Figure 6B), and tended to be inversely related to IL‐10 concentrations, although this was not statistically supported.

We next studied correlations between the HA‐IgG AUC and HI‐responses with cytokine concentrations. There was a significant inverse correlation between baseline IL‐10 concentrations and G1.AUC in the pooled analyses (Figure 6C), although no significant correlations were found in the subgroup analyses, likely due to the loss of statistical power (Figure S2). When we examined the reactivity profile of individuals with detectable IL‐10 versus those with IL‐10 concentrations that were LLoQ (IL‐10‐negative), there was an association between the IL‐10‐negative individuals and Cluster 1 (p = 0.054, Figure 6D). No clear relationship was observed between IL‐10 positivity and titer‐fold changes (Figure 6E). TNF‐α concentrations were positively correlated with HI‐fold change titers for subtype H1N1 in HRs (p = 0.03, Figure 6E), likely due to the larger effect size in the H1N1 HI‐response. Taken together, LRs had higher concentrations of IL‐10 at vaccination, which was also correlated with lower titers of Group 1 HA‐IgG antibodies, and concentrations of TNF‐α were positively correlated with HI‐responses in HRs.

## 4 DISCUSSION

In order to better understand the underlying causes of poor influenza vaccine efficacy in older adults, we used HA‐IgG binding profiles against a panel of HAs to capture the dynamic immunological changes more broadly before and after inactivated influenza vaccination. We found that, on an aggregated level, LRs responded better to the H3N2 vaccine component compared with the H1N1 vaccine component, which was also associated with less total Group 1 HA‐IgG at baseline. We also found that LRs had, on average, higher concentrations of IL‐10 at baseline that negatively correlated to total Group 1 HA‐IgG at baseline.

Our findings suggest that host inflammatory status and pre‐existing influenza immunity can impact influenza vaccine responses through different mechanisms. The older poor vaccine responders may be immunologically restricted in their ability to generate vaccine‐specific HI‐responses, but pre‐existing influenza‐specific B cells can still be activated to produce IgG antibodies,^14^, ^24^ although this boosting appears to have a threshold. The magnitude of IgG‐titer increases in HRs was inversely correlated with pre‐existing titers, reflecting the “antibody ceiling effect” previously reported for HI and IgG responses during infection^24^, ^25^ and vaccination.^13^, ^16^, ^26^, ^27^ High titers of pre‐existing antibodies could lead to more rapid antigen clearance,^28^, ^29^ leading to limited activation of pre‐existing CD4+ T cells and memory B cells.^24^ However, a ceiling effect for IgG responses was not detrimental since HRs, in which it was more evident, were still capable of mounting robust HI‐antibody responses. An alternative explanation to the “ceiling effect” is that the kinetics of a “boosted” IgG‐antibody response was more rapid and had subsided by the time that is typically used to measure a vaccine‐induced antibody response using HI‐assays.^30^

Our data suggests that host inflammatory status may be a more important driver in generating HI‐responses after vaccination compared with preexisting influenza immunity in older adults. As with previous studies, IL‐6 and TNF‐α concentrations were positively correlated with each other,^8^, ^31^ and higher TNF‐α concentrations were associated with a greater magnitude of HI antibody response in the HRs. In contrast, IL‐10 was significantly higher in the LRs compared to the HRs. This was also previously reported for hepatitis B with the live‐attenuated zoster vaccines.^32^, ^33^ Notably, similar to our findings, the induction of antibodies measured by the fluorescence‐antibody‐to‐membrane‐antigen (FAMA) assay, which are associated with protection, was particularly inhibited compared with the antibodies measured by IgG‐ELISA. Like the influenza HI‐assay, the FAMA assay uses viral surface antigens that are conformationally specific and intact and therefore measures antibodies that have virus neutralization potential. IgG‐ELISA, in contrast, uses antigens that are non‐conformationally specific and thus detects all classes of antibodies that recognize the protein, offering assay sensitivity but not necessarily functional specificity.

One possible process that may be affected by the high levels of IL‐10 is the antigen presentation pathway. Mohanty et al. showed that monocytes isolated from older adults secrete more IL‐10 compared to younger adults. Crucially, when these data were parsed into vaccine responders and non‐responders, induction of IL‐6 and TNF‐α was associated with vaccine responders regardless of age, while the inhibitory effects of IL‐10 appeared to be specific only to the older non‐responders.^34^ Another study in aged mice suggested that IL‐10 could also be produced by T‐follicular helper cells, a cell population critical for germinal center responses that generate durable and high‐affinity antibodies.^35^ Collectively, these and our study suggest that an IL‐10‐associated impairment of antigen presentation and germinal center responses could contribute to the lack of post‐vaccination HI‐antibody response seen in older LRs.

Since subtype H3N2 dominated for six of the 10 influenza seasons between 2010 and 2020 in China,^36^ the high H3 IAV baseline titers may have been boosted by recent infections. It is unclear whether the low Group 1 reactivity in LRs was simply a lack of recent exposure or indicative of immunological deficits in sustaining a memory response to H1N1 IAVs. Both scenarios are plausible within the context of our current understanding of immunosenescence^3^ and should be tested in further studies. Further, we also found no clear evidence of immunological imprinting based on the HA‐IgG response.

There were some limitations to our study. Our study was correlative, and there may be other as yet unidentified factors leading to the poor vaccine responses in LRs. Furthermore, our sample sizes may have limited statistical power to detect other associations between cytokine concentrations and antibody responses. The cytokine milieu is complex, and the effects of IL‐10 may be antagonized by other pro‐inflammatory cytokines post‐vaccination. For example, the ratio of IFN‐γ and IL‐10 secreted by immune cells collected after influenza vaccination, rather than IL‐10 levels alone, was found to be associated with protection.^37^

To conclude, we found that the LRs in our cohort were not necessarily unresponsive to the vaccine since they made comparable titers of H3‐IgG antibodies to HRs. This occurred for the H3 but not the H1 vaccine components, suggesting that high pre‐existing H3 immunity was likely a contributor that may also contribute to the protective effects of repeated influenza vaccination in older adults.^38^ The inability to elicit robust HI‐antibodies was potentially associated with elevated levels of IL‐10 at the time of vaccination. Our findings highlight the diverse mechanisms impacting influenza vaccine responses in older adults and suggest that a better understanding of prior influenza immunity that is not limited to HI‐responses could help clarify the heterogeneity observed in the field. Finally, the potential for IL‐10 as a biomarker for poor vaccine responders in older adults has translational potential and should be explored further.

## AUTHOR CONTRIBUTIONS

*Study conceptualization*: Sook‐San Wong, Shixing Tang, Min Kang, and Mark Zanin. *Performed the experiments and collected data*: Fangmei Lin, Xiaohua Tan, Zaolan Liang, and Xia Lin. *Data analysis*: Fangmei Lin, Yonghe Xia, Wenda Guan, Zifeng Yang, and Zhanpeng Jiang. *Statistics and landscape generation*: Yonghe Xia and Guangchuang Yu. *Manuscript preparation*: Fangmei Lin, Zhanpeng Jiang, Sook‐San Wong, Min Kang, and Mark Zanin. *Final draft*: Sook‐San Wong, Shixing Tang, Min Kang, and Mark Zanin. *Funding acquisition*: Min Kang, Shixing Tang, and Sook‐San Wong. All authors have read and approved the final manuscript.

## CONFLICT OF INTEREST STATEMENT

Min Kang and Sook‐San Wong have previously received speakers' honoraria from Sanofi‐Pasteur (China). All other authors declare no conflicts of interest.

### PEER REVIEW

The peer review history for this article is available at https://www.webofscience.com/api/gateway/wos/peer-review/10.1111/irv.13172.

## Supporting information


**Figure S1.** Determination of optimal number of clusters for k‐means clustering. (A) Results from NBClust [R1]. (B) Resolution of data points when k = 2, 3 and 4 were used in the K‐means clustering analysis.
**Figure S2.** Correlation matrix for the cytokine concentrations with the HA‐IgG profile based on the Group 1 (G1) and Group 2 (G2) AUC at Day 0 (D0) and Day 30 (D30) and its’ respective fold‐changes (FOLD) for (A) low and (B) high responders. One low responder with outlying IL‐10 signal was excluded from analysis. Color scale and the size of the circle in (B to D) are proportional to the Pearson’s correlation and the r‐values, respectively.
**Table S1.** Antibody responses to the influenza A vaccine components in the high and low‐responders as measured by neuraminidase‐inhibition (NI) assay. Antibody titers were expressed as geometric mean titers (GMT) and fold‐changes between Day 0 (GMT 1) and Day 30 (GMT 2) were expressed as Geometric Mean Fold Change (GMFC). Seroconversions (SC) refers to percentage of vaccinees that achieved at least a four‐fold increase in antibody titers after vaccination at Day 30. Statistically significant p‐values (<0.05), adjusted for multiple comparisons, are italicized while ns denotes no significance.
**Table S2.** Antibody responses to the influenza A vaccine components in the high and low‐responders as measured by HA‐IgG ELISA. Antibody titers were expressed as geometric mean titers (GMT) and fold‐changes between Day 0 (GMT 1) and Day 30 (GMT 2) were expressed as Geometric Mean Fold Change (GMFC). Statistically significant p‐values (<0.05), adjusted for multiple comparisons, are italicized while ns denotes no significance.
**Table S3.** List of recombinant HA protein used in the ELISA. All proteins were expressed with polyhistidine (HIS) tags in human embryonic kidney (HEK) 293 cells and purchased from Sinobiological Inc (Beijing, China). Vaccine strains are indicated with asterisks.
**Table S4.** Raw data of the cytokine concentrations as determined by the bead‐based assay. All concentrations are pg/ml. Values that are lower than the lowest point on the standard curve are highlighted in green (limit of quantification, LoQ), while values that are lower than the sensitivity of the assay are highlighted in red. Note that the IL‐10 threshold for LoQ and assay sensitivity are the same. Values that were lower than the LoQ or assay sensitivity were assigned the median of the range of lower than LoQ values. * indicate the outlier that was excluded from Figure 6B.

## Data Availability

Data are available from the first or corresponding authors upon request.
