# Supplementary material for: The impact of pre‐existing influenza antibodies and inflammatory status on the influenza vaccine responses in older adults
Source: Influenza Other Respir Viruses. 2023 Jul 12;17(7):e13172. doi: 10.1111/irv.13172 (PMC10339007; doi:10.1111/irv.13172)
Supplement: Supplementary file 1 — Figure S1. Determination of optimal number of clusters for k‐means clustering. (A) Results from NBClust [R1]. (B) Resolution of data points when k = 2, 3 and 4 were used in the K‐means clustering analysis. Figure S2. Correlation matrix for the cytokine concentrations with the HA‐IgG profile based on the Group 1 (G1) and Group 2 (G2) AUC at Day 0 (D0) and Day 30 (D30) and its’ respective fold‐changes (FOLD) for (A) low and (B) high responders. One low responder with outlying IL‐10 signal was excluded from analysis. Color scale and the size of the circle in (B to D) are proportional to the Pearson’s correlation and the r‐values, respectively. Table S1. Antibody responses to the influenza A vaccine components in the high and low‐responders as measured by neuraminidase‐inhibition (NI) assay. Antibody titers were expressed as geometric mean titers (GMT) and fold‐changes between Day 0 (GMT 1) and Day 30 (GMT 2) were expressed as Geometric Mean Fold Change (GMFC). Seroconversions (SC) refers to percentage of vaccinees that achieved at least a four‐fold increase in antibody titers after vaccination at Day 30. Statistically significant p‐values (<0.05), adjusted for multiple comparisons, are italicized while ns denotes no significance. Table S2. Antibody responses to the influenza A vaccine components in the high and low‐responders as measured by HA‐IgG ELISA. Antibody titers were expressed as geometric mean titers (GMT) and fold‐changes between Day 0 (GMT 1) and Day 30 (GMT 2) were expressed as Geometric Mean Fold Change (GMFC). Statistically significant p‐values (<0.05), adjusted for multiple comparisons, are italicized while ns denotes no significance. Table S3. List of recombinant HA protein used in the ELISA. All proteins were expressed with polyhistidine (HIS) tags in human embryonic kidney (HEK) 293 cells and purchased from Sinobiological Inc (Beijing, China). Vaccine strains are indicated with asterisks. Table S4. Raw data of the cytokine concentrations as determined [file IRV-17-e13172-s001.docx]

**Supplemental Material: The impact of pre-existing influenza antibodies and inflammatory status on the influenza vaccine responses in older adults.**

**Supplemental Figure 1.** Determination of optimal number of clusters for k-means clustering. (A) Results from NBClust [R1]. (B) Resolution of data points when k=2, 3 and 4 were used in the K-means clustering analysis.

**A**

**B**

**Supplemental Figure 2.** Correlation matrix for the cytokine concentrations with the HA-IgG profile based on the Group 1 (G1) and Group 2 (G2) AUC at Day 0 (D0) and Day 30 (D30) and its’ respective fold-changes (FOLD) for (A) low and (B) high responders. One low responder with outlying IL-10 signal was excluded from analysis. Color scale and the size of the circle in (B to D) are proportional to the Pearson’s correlation and the r-values, respectively.

**B**

**A**

**Supplemental Table 1.** Antibody responses to the influenza A vaccine components in the high and low-responders as measured by neuraminidase-inhibition (NI) assay. Antibody titers were expressed as geometric mean titers (GMT) and fold-changes between Day 0 (GMT 1) and Day 30 (GMT 2) were expressed as Geometric Mean Fold Change (GMFC). Seroconversions (SC) refers to percentage of vaccinees that achieved at least a four-fold increase in antibody titers after vaccination at Day 30. Statistically significant p-values (<0.05), adjusted for multiple comparisons, are italicized while ns denotes no significance.

| **Groups** | **Antibody titer** | **N** | **N1** | | | **N2** | | |
| --- | --- | --- | --- | --- | --- | --- | --- | --- |
|  |  |  | **NI** | **95%CI** | **p-value** | **NI** | **95%CI** | **p-value** |
| Low | GMT 1 | 29 | 43 | 29.5-62.5 |  | 26 | 18.6-36.3 |  |
|  | GMT 2 |  | 63 | 39.2-101.2 | 0.062^a^ | 39 | 25.2-60.5 | 0.088^a^ |
|  | GMFC |  | 1.5 | 1.0-2.2 |  | 1.5 | 0.9-2.4 |  |
|  | %SC |  | 34 |  |  | 24 |  |  |
| High | GMT 1 | 26^^^ | 72 | 40.3-128.5 | ns^c^ | 31 | 20.9-47.4 | ns^c^ |
|  | GMT 2 |  | 215 | 129.1-356.6 | *0.006*^b^, *0.0008*^d^ | 63 | 38.2-103.8 | *0.0054*^b^, ns^d^ |
|  | GMFC |  | 3.0 | 1.4-6.3 | 0.081^e^ | 2.0 | 1.3-3.2 | 0.380 ^e^ |
|  | %SC |  | 50 |  |  | 35 |  |  |

^^^ Four samples did not have sufficient sera for testing.

^a^ GMT 2 versus GMT 1 by t-test in low-responders.

^b^ GMT 2 versus GMT 1 by t-test in high-responders.

^c^ GMT 1, High vs Low by t-test using log-transformed data.

^d^ GMT 2, High vs Low by t-test using log-transformed data.

^e^ GMFC, High vs Low by t-test using log-transformed data.

**Supplemental Table 2.** Antibody responses to the influenza A vaccine components in the high and low-responders as measured by HA-IgG ELISA. Antibody titers were expressed as geometric mean titers (GMT) and fold-changes between Day 0 (GMT 1) and Day 30 (GMT 2) were expressed as Geometric Mean Fold Change (GMFC). Statistically significant p-values (<0.05), adjusted for multiple comparisons, are italicized while ns denotes no significance.

| **Groups** | **Antibody titer** | **N** | **A/Michigan/45/2015 (H1N1)** | | |  | **A/Singapore/INFIMH160019/2016 (H3N2)** | | |
| --- | --- | --- | --- | --- | --- | --- | --- | --- | --- |
|  |  |  | **HA-IgG** | **95%CI** | **p-value** |  | **HA-IgG** | **95%CI** | **p-value** |
| Low | GMT 1 | 29 | 3.337 | 3.1-3.7 |  |  | 67.67 | 56.4-81.1 |  |
|  | GMT 2 |  | 3.651 | 3.4-3.9 | 0.097^a^ |  | 81.94 | 66.7-100.6 | *0.009^a^* |
|  | GMFC |  | 1.953 | 0.9-4.3 |  |  | 2.42 | 1.3-4.6 |  |
| High | GMT 1 | 26^^^ | 3.673 | 3.4-4.0 | ns^c^ |  | 54.01 | 41.6-70.1 | ns^c^ |
|  | GMT 2 |  | 4.425 | 4.2-4.7 | *<0.001^b^, 0.0002*^d^ |  | 412.60 | 278.4-611.4 | *0.03^b^,* ns^d^ |
|  | GMFC |  | 5.040 | 2.3-11.2 | 0.090^e^ |  | 1.95 | 1.1-3.6 | 0.619^e^ |

^^^ Four samples did not have sufficient sera for testing.

^a^ GMT 2 versus GMT 1 by t-test in low-responders.

^b^ GMT 2 versus GMT 1 by t-test in high-responders.

^c^ GMT 1, High vs Low by t-test using log-transformed data.

^d^ GMT 2, High vs Low by t-test using log-transformed data.

^e^ GMFC, High vs Low by t-test using log-transformed data.

**Supplemental Table 3.** List of recombinant HA protein used in the ELISA. All proteins were expressed with polyhistidine (HIS) tags in human embryonic kidney (HEK) 293 cells and purchased from Sinobiological Inc (Beijing, China). Vaccine strains are indicated with asterisks.

| **No.** | **Group** | **Subtype** | **Strain** | **Catalog Number** | **Abbr.** |
| --- | --- | --- | --- | --- | --- |
| 1 | 1 | H1N1 | A/Brevig Mission/1/1918 | 11068-V08H | H1.BM18 |
| 2 | 1 | H1N1 | A/Puerto Rico/8/34 | 11684-V08H | H1.PR34 |
| 3 | 1 | H1N1 | A/New Caledonia/20/1999 | 11683-V08H | H1.NC99 |
| 3 | 1 | H1N1 | A/Brisbane/59/2007 | 11052-V08H | H1.BN07 |
| 4 | 1 | H1N1 | A/California/07/2009 | 11085-V08H | H1.CA09 |
| 5 | 1 | H1N1 | A/Michigan/45/2015* | 40567-H08H | H1.MI15 |
| 6 | 1 | H2N2 | A/Japan/305/1957 | 11088-V08H | H2 |
| 7 | 1 | H5N3 | A/duck/Hokkaido/167/2007 | 11696-V08H | H5.Dk07 |
| 8 | 1 | H5N1 | A/Hubei/1/2010 | 40015-V08H | H5.HU10 |
| 9 | 1 | H6N4 | A/chicken/Hong Kong/17/1977 | 40027-V08H | H6 |
| 10 | 1 | H9N2 | A/Hong Kong/1073/99 | 11229-V08H | H9 |
| 11 | 1 | H11N2 | A/duck/Yangzhou/906/2002 | 11705-V08H | H11 |
| 12 | 1 | H12N5 | A/green-winged teal/ALB/199/1991 | 11718-V08H | H12 |
| 13 | 1 | H13N8 | A/black-headed gull/Netherlands/1/00 | 11721-V08H | H13 |
| 14 | 1 | H16N3 | A/black-headed gull/Sweden/5/99 | 11711-V08H | H16 |
| 15 | 2 | H3N2 | A/Aichi/2/1968 | 11707-V08H | H3.68 |
| 16 | 2 | H3N2 | A/Perth/16/2009 | 40043-V08H | H3.09 |
| 17 | 2 | H3N2 | A/HongKong/4801/2014 | 40555-V08B | H3.HK14 |
| 18 | 2 | H3N2 | A/Singapore/INFIMH160019/2016* | 40580-V08H | H3.SG16 |
| 19 | 2 | H4N6 | A/mallard/Ohio/657/2002 | 11714-V08H | H4 |
| 20 | 2 | H7N9 | A/Anhui/1/2013 | 40103-V08H | H7.AN13 |
| 21 | 2 | H7N7 | A/Netherlands/219/2003 | 11082-V08B | H7.NL03 |
| 22 | 2 | H7N7 | A/equine/Kentucky/1a/1975 | 40171-V08B | H7.Eq75 |
| 23 | 2 | H10N3 | A/duck/Hong Kong/786/1979 | 11693-V08H | H10 |

**Supplemental Table 4.** Raw data of the cytokine concentrations as determined by the bead-based assay. All concentrations are pg/ml. Values that are lower than the lowest point on the standard curve are highlighted in green (limit of quantification, LoQ), while values that are lower than the sensitivity of the assay are highlighted in red. Note that the IL-10 threshold for LoQ and assay sensitivity are the same. Values that were lower than the LoQ or assay sensitivity were assigned the median of the range of lower than LoQ values. * indicate the outlier that was excluded from Figure 6B.

| **Sample ID** | **TNF-α** | **IL-6** | **IL-10** | **IFN-γ** |
| --- | --- | --- | --- | --- |
| Assay sensitivity | 0.54 | 0.31 | 0.24 | 0.25 |
| Range of standard curve | 0.757-3100 | 0.854-3500 | 0.244-1000 | 3.71-15200 |
| 1 | 4.83 | 1.96 | 0.01 | 2.11 |
| 2 | 4.83 | 0.86 | 0.01 | 1.58 |
| 3 | 2.6 | 0.71 | 0.03 | 1.74 |
| 4 | 0.88 | 0.84 | 0.16 | 2.16 |
| 5 | 3.67 | 1.31 | 0.17 | 2 |
| 6 | 4.27 | 0.97 | 0.23 | 1.95 |
| 7 | 3.67 | 1.5 | 0.28 | 2.55 |
| 8 | 0.63 | 0.84 | 0.35 | 2.33 |
| 9 | 5.58 | 0.97 | 0.37 | 2.27 |
| 10 | 0.79 | 0.37 | 0.48 | 2 |
| 11 | 8.09 | 2.27 | 0.51 | 2.11 |
| 12 | 2.22 | 0.46 | 0.51 | 2 |
| 13 | 14.83 | 5.97 | 0.59 | 2.44 |
| 14 | 7.11 | 0.71 | 0.59 | 2.33 |
| 15 | 7.58 | 0.61 | 0.64 | 2.11 |
| 16 | 4.38 | 0.86 | 0.77 | 2 |
| 17 | 9.21 | 1.34 | 0.77 | 3.53 |
| 18 | 5.45 | 1.81 | 0.77 | 2 |
| 19 | 9.01 | 1.23 | 0.94 | 2.22 |
| 20 | 5.58 | 0.61 | 0.94 | 2.06 |
| 21 | 3.49 | 1.29 | 1.04 | 2.11 |
| 22 | 0.71 | 0.56 | 1.16 | 2.11 |
| 23 | 0.63 | 0.71 | 1.16 | 2.33 |
| 24 | 0.52 | 0.71 | 1.16 | 1.9 |
| 25 | 4.27 | 3.63 | 1.22 | 1.9 |
| 26 | 4.72 | 0.97 | 2.24 | 2.11 |
| 27 | 0.56 | 0.81 | 2.66 | 2.16 |
| 28 | 18.01 | 17.73 | 69.82* | 2.77 |
| 29 | NA | NA | NA | NA |
| 30 | 6.38 | 2.27 | 0.005 | 2.55 |
| 31 | 4.38 | 0.61 | 0.005 | 2.55 |
| 32 | 2.66 | 0.86 | 0.005 | 2 |
| 33 | 5.58 | 1.07 | 0.005 | 2.11 |
| 34 | 1.86 | 2.42 | 0.005 | 5.38 |
| 35 | 1.64 | 26.75 | 0.01 | 1.9 |
| 36 | 6.52 | 1.45 | 0.03 | 2.55 |
| 37 | 12.2 | 2.77 | 0.03 | 2 |
| 38 | 7.92 | 7.62 | 0.05 | 2.11 |
| 39 | 9.83 | 0.86 | 0.06 | 1.84 |
| 40 | 10.04 | 0.97 | 0.09 | 1.95 |
| 41 | 10.04 | 1.81 | 0.12 | 2.44 |
| 42 | 6.95 | 2.91 | 0.13 | 2 |
| 43 | 7.58 | 0.94 | 0.22 | 2 |
| 44 | 0.71 | 0.61 | 0.22 | 1.58 |
| 45 | 2.73 | 0.53 | 0.27 | 1.69 |
| 46 | 0.79 | 0.46 | 0.27 | 2.33 |
| 47 | 0.56 | 0.63 | 0.33 | 2.22 |
| 48 | 0.63 | 0.61 | 0.34 | 2.22 |
| 49 | 12.47 | 0.71 | 0.35 | 2.49 |
| 50 | 0.63 | 1.15 | 0.44 | 1.38 |
| 51 | 13.02 | 2.77 | 0.55 | 2.22 |
| 52 | 3.86 | 4.02 | 0.77 | 2.22 |
| 53 | 11.69 | 1.67 | 0.85 | 2.66 |
| 54 | 0.45 | 0.71 | 0.85 | 1.95 |
| 55 | 4.6 | 0.81 | 1.04 | 2.06 |
| 56 | 0.83 | 0.81 | 1.04 | 1.58 |
| 57 | 0.6 | 0.56 | 1.42 | 1.79 |
| 58 | NA | NA | NA | NA |
| 59 | NA | NA | NA | NA |
